# *catena*-Poly[[[diaqua­diformatonickel(II)]-μ-1,4-bis­(1*H*-benzimidazol-1-yl)benzene] dihydrate]

**DOI:** 10.1107/S160053681200284X

**Published:** 2012-01-31

**Authors:** Hui Li, Hong Sun, Xiaochuan Chai, Chenzhong Yao

**Affiliations:** a Department of Applied Chemistry, Yuncheng University, Yuncheng, Shanxi 044000, People’s Republic of China

## Abstract

In the title one-dimensional coordination polymer, {[Ni(CHO_2_)_2_(C_20_H_14_N_4_)(H_2_O)_2_]·2H_2_O}_*n*_, the Ni^II^ atom lies on a crystallographic inversion centre. It is coordinated by two formate O atoms, two water O atoms and two N atoms from two 1,4-bis­(1*H*-benzimidazol-1-yl)benzene (bzb) ligands, resulting in a distorted *trans*-NiN_2_O_4_ octa­hedral coordination geometry. The bzb mol­ecule acts as a bridging ligand to connect the metal atoms into a chain propagating in [1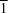 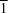]. The dihedral angle between the benzimidazole ring and the central benzene ring in the ligand is 38.16 (9)°. In the crystal, O—H⋯O hydrogen bonds crosslink the chains into (010) sheets.

## Related literature

For background to coordination polymers containing imidazole-derived ligands, see: Li *et al.* (2009 ▶, 2011 ▶).
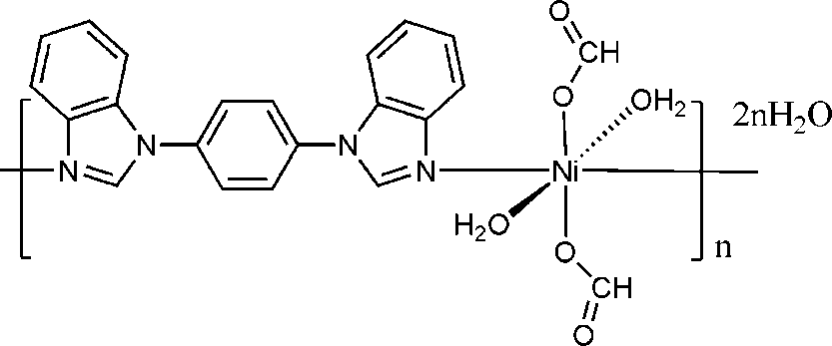


## Experimental

### 

#### Crystal data

[Ni(CHO_2_)_2_(C_20_H_14_N_4_)(H_2_O)_2_]·2H_2_O
*M* *_r_* = 531.16
Triclinic, 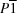

*a* = 7.4431 (15) Å
*b* = 9.0895 (18) Å
*c* = 9.3863 (19) Å
α = 78.46 (3)°
β = 77.79 (3)°
γ = 67.86 (3)°
*V* = 569.8 (2) Å^3^
*Z* = 1
Mo *K*α radiation
μ = 0.91 mm^−1^
*T* = 293 K
0.25 × 0.22 × 0.20 mm

#### Data collection

Rigaku Mercury CCD diffractometer
Absorption correction: multi-scan (*CrystalClear*; Rigaku/MSC, 2005 ▶) *T*_min_ = 0.797, *T*_max_ = 0.834
5022 measured reflections
2000 independent reflections
1874 reflections with *I* > 2σ(*I*)
*R*_int_ = 0.021

#### Refinement

*R*[*F* ^2^ > 2σ(*F* ^2^)] = 0.027
*wR*(*F* ^2^) = 0.061
*S* = 1.09
2000 reflections
160 parameters
H-atom parameters constrained
Δρ_max_ = 0.23 e Å^−3^
Δρ_min_ = −0.19 e Å^−3^

### 

Data collection: *CrystalClear* (Rigaku/MSC, 2005 ▶); cell refinement: *CrystalClear*; data reduction: *CrystalClear*; program(s) used to solve structure: *SHELXS97* (Sheldrick, 2008 ▶); program(s) used to refine structure: *SHELXL97* (Sheldrick, 2008 ▶); molecular graphics: *SHELXTL* (Sheldrick, 2008 ▶); software used to prepare material for publication: *SHELXTL*.

## Supplementary Material

Crystal structure: contains datablock(s) I, global. DOI: 10.1107/S160053681200284X/hb6604sup1.cif

Structure factors: contains datablock(s) I. DOI: 10.1107/S160053681200284X/hb6604Isup2.hkl

Additional supplementary materials:  crystallographic information; 3D view; checkCIF report

## Figures and Tables

**Table 1:** Selected bond lengths (Å)

Ni1—O1	2.0695 (14)
Ni1—N1	2.0908 (16)
Ni1—O1*W*	2.1036 (16)

**Table 2:** Hydrogen-bond geometry (Å, °)

*D*—H⋯*A*	*D*—H	H⋯*A*	*D*⋯*A*	*D*—H⋯*A*
O1*W*—H1*A*⋯O2^i^	0.85	1.85	2.694 (2)	169
O1*W*—H1*B*⋯O2*W*^ii^	0.85	1.92	2.762 (2)	169
O2*W*—H2*A*⋯O2^iii^	0.85	1.91	2.760 (2)	173
O2*W*—H2*B*⋯O1^iv^	0.85	2.16	2.846 (2)	137

Symmetry codes: (i) 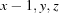; (ii) 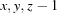; (iii) 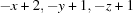; (iv) 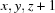.

**Fig. 1.**
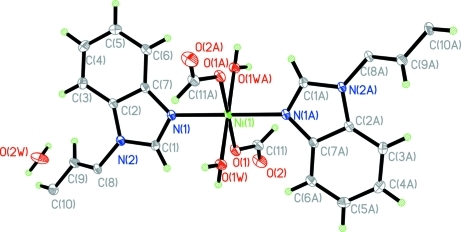
The molecular structure of (I). Displacement ellipsoids are drawn at the 30% probability level and H atoms are shown as small spheres of arbitrary radius.

**Fig. 2.**
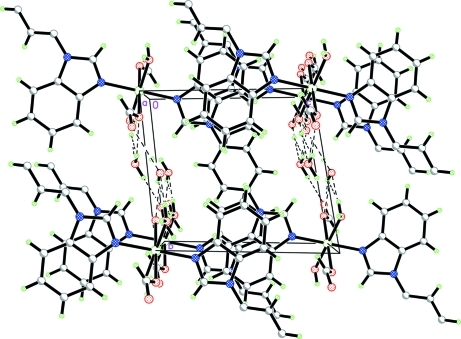
The crystal packing for (I).

## supplementary crystallographic information

### Comment

Imidazole has been extensively used in crystal engineering, and a large number of imidazole-containing flexible ligands have been extensively studied. However, to our knowledge, the research on imidazole ligands bearing rigid spacers is still less developed (Li *et al.*, 2009; Li *et al.*, 2011). For the title compound, the geometry of the Ni^II^ ion is bound by two benzimidazole rings of individual L ligands, two water molecules and two formate ions forming a slightly distorted octahedral coordination environment(Fig. 1). Notably, as shown in Fig. 2, the six-coordinate Ni^II^ center is bridged by the ligand L to form an infinite one-dimensional architecture.

### Experimental

A mixture of CH_3_OH and H_2_O (1:1, 8 ml), as a buffer layer, was carefully layered over a solution of Ni(HCO_2_)_2_ in H_2_O (6 ml). Then a solution of 1,4-di(1*H*-benzimidazol-1-yl)benzene (L, 0.06 mmol) in CH_3_OH (6 ml) was layered over the buffer layer, and the resultant reaction was left to stand at room temperature. After *ca* three weeks, green block single crystals appeared at the boundary. Yield: ~20% (based on L).

### Refinement

C-bound H atoms were positioned geometrically and refined in the riding-model approximation, with C—H = 0.93Å and *U*_iso_(H) = 1.2*U*_eq_ (C).

### Crystal data

**Table:**

[Ni(CHO_2_)_2_(C_20_H_14_N_4_)(H_2_O)_2_]·2H_2_O	*Z* = 1
*M**_r_* = 531.16	*F*(000) = 276
Triclinic, *P*1	*D*_x_ = 1.548 Mg m^−^^3^
Hall symbol: -p 1	Mo *K*α radiation, λ = 0.71073 Å
*a* = 7.4431 (15) Å	Cell parameters from 6111 reflections
*b* = 9.0895 (18) Å	θ = 6.2–55.0°
*c* = 9.3863 (19) Å	µ = 0.91 mm^−^^1^
α = 78.46 (3)°	*T* = 293 K
β = 77.79 (3)°	Block, green
γ = 67.86 (3)°	0.25 × 0.22 × 0.20 mm
*V* = 569.8 (2) Å^3^	

### Data collection

**Table:**

Rigaku Mercury CCD diffractometer	2000 independent reflections
Radiation source: fine-focus sealed tube	1874 reflections with *I* > 2σ(*I*)
graphite	*R*_int_ = 0.021
Detector resolution: 9 pixels mm^-1^	θ_max_ = 25.0°, θ_min_ = 3.1°
ω scans	*h* = −8→8
Absorption correction: multi-scan (*CrystalClear*; Rigaku/MSC, 2005)	*k* = −10→10
*T*_min_ = 0.797, *T*_max_ = 0.834	*l* = −11→11
5022 measured reflections	

### Refinement

**Table:**

Refinement on *F*^2^	Primary atom site location: structure-invariant direct methods
Least-squares matrix: full	Secondary atom site location: difference Fourier map
*R*[*F*^2^ > 2σ(*F*^2^)] = 0.027	Hydrogen site location: inferred from neighbouring sites
*wR*(*F*^2^) = 0.061	H-atom parameters constrained
*S* = 1.09	*w* = 1/[σ^2^(*F*_o_^2^) + (0.0208*P*)^2^ + 0.3422*P*] where *P* = (*F*_o_^2^ + 2*F*_c_^2^)/3
2000 reflections	(Δ/σ)_max_ < 0.001
160 parameters	Δρ_max_ = 0.23 e Å^−^^3^
0 restraints	Δρ_min_ = −0.19 e Å^−^^3^

### Special details

**Table:**

Geometry. All e.s.d.'s (except the e.s.d. in the dihedral angle between two l.s. planes) are estimated using the full covariance matrix. The cell e.s.d.'s are taken into account individually in the estimation of e.s.d.'s in distances, angles and torsion angles; correlations between e.s.d.'s in cell parameters are only used when they are defined by crystal symmetry. An approximate (isotropic) treatment of cell e.s.d.'s is used for estimating e.s.d.'s involving l.s. planes.
Refinement. Refinement of *F*^2^ against ALL reflections. The weighted *R*-factor *wR* and goodness of fit *S* are based on *F*^2^, conventional *R*-factors *R* are based on *F*, with *F* set to zero for negative *F*^2^. The threshold expression of *F*^2^ > σ(*F*^2^) is used only for calculating *R*-factors(gt) *etc*. and is not relevant to the choice of reflections for refinement. *R*-factors based on *F*^2^ are statistically about twice as large as those based on *F*, and *R*- factors based on ALL data will be even larger.

### Fractional atomic coordinates and isotropic or equivalent isotropic displacement parameters (Å^2^)

**Table:**

	*x*	*y*	*z*	*U*_iso_*/*U*_eq_	
Ni1	1.0000	0.0000	0.0000	0.01789 (11)	
N1	0.8599 (2)	0.05059 (18)	0.21160 (16)	0.0233 (4)	
N2	0.7076 (2)	0.21017 (19)	0.38522 (17)	0.0251 (4)	
O1	1.11753 (19)	0.17566 (16)	−0.00983 (15)	0.0272 (3)	
O2	1.3775 (2)	0.24681 (19)	−0.03883 (19)	0.0439 (4)	
O2W	0.8143 (3)	0.4708 (2)	0.9185 (2)	0.0580 (5)	
O1W	0.76721 (19)	0.18582 (16)	−0.09086 (15)	0.0265 (3)	
C1	0.7822 (3)	0.1980 (2)	0.2416 (2)	0.0265 (4)	
H1	0.7785	0.2864	0.1709	0.032*	
C2	0.7415 (3)	0.0544 (2)	0.4562 (2)	0.0243 (4)	
C3	0.6960 (3)	−0.0069 (3)	0.6010 (2)	0.0349 (5)	
H3	0.6319	0.0598	0.6733	0.042*	
C4	0.7496 (4)	−0.1702 (3)	0.6330 (2)	0.0410 (6)	
H4	0.7235	−0.2156	0.7294	0.049*	
C5	0.8426 (3)	−0.2698 (3)	0.5238 (3)	0.0390 (5)	
H5	0.8762	−0.3800	0.5493	0.047*	
C6	0.8858 (3)	−0.2091 (2)	0.3798 (2)	0.0301 (5)	
H6	0.9465	−0.2762	0.3076	0.036*	
C7	0.8359 (3)	−0.0440 (2)	0.3458 (2)	0.0223 (4)	
C8	0.6026 (3)	0.3572 (2)	0.4442 (2)	0.0242 (4)	
C9	0.6216 (3)	0.3723 (2)	0.5828 (2)	0.0280 (5)	
H9	0.7031	0.2864	0.6386	0.034*	
C10	0.5193 (3)	0.5154 (2)	0.6388 (2)	0.0292 (5)	
H10	0.5321	0.5262	0.7322	0.035*	
C11	1.2948 (3)	0.1551 (2)	−0.0493 (2)	0.0280 (5)	
H11	1.3723	0.0622	−0.0905	0.034*	
H1A	0.6479	0.1921	−0.0709	0.042*	
H1B	0.7658	0.2799	−0.0905	0.042*	
H2A	0.7644	0.5582	0.9559	0.042*	
H2B	0.9293	0.4212	0.9405	0.042*	

### Atomic displacement parameters (Å^2^)

**Table:**

	*U*^11^	*U*^22^	*U*^33^	*U*^12^	*U*^13^	*U*^23^
Ni1	0.01748 (19)	0.01802 (19)	0.01746 (19)	−0.00478 (14)	0.00105 (13)	−0.00749 (13)
N1	0.0272 (9)	0.0210 (9)	0.0184 (8)	−0.0052 (7)	0.0011 (7)	−0.0064 (7)
N2	0.0323 (9)	0.0199 (8)	0.0185 (8)	−0.0045 (7)	0.0018 (7)	−0.0073 (6)
O1	0.0218 (7)	0.0261 (8)	0.0348 (8)	−0.0090 (6)	0.0019 (6)	−0.0117 (6)
O2	0.0276 (8)	0.0366 (9)	0.0723 (12)	−0.0153 (7)	−0.0012 (8)	−0.0161 (8)
O2W	0.0580 (12)	0.0302 (9)	0.0900 (15)	−0.0068 (8)	−0.0309 (10)	−0.0132 (9)
O1W	0.0220 (7)	0.0249 (7)	0.0311 (8)	−0.0061 (6)	−0.0041 (6)	−0.0041 (6)
C1	0.0348 (11)	0.0218 (10)	0.0185 (10)	−0.0068 (9)	0.0017 (8)	−0.0052 (8)
C2	0.0247 (10)	0.0217 (10)	0.0230 (10)	−0.0043 (8)	0.0000 (8)	−0.0067 (8)
C3	0.0440 (13)	0.0329 (12)	0.0215 (11)	−0.0100 (10)	0.0042 (9)	−0.0063 (9)
C4	0.0539 (15)	0.0345 (13)	0.0257 (12)	−0.0133 (11)	0.0021 (10)	0.0031 (9)
C5	0.0459 (14)	0.0235 (11)	0.0397 (13)	−0.0089 (10)	0.0003 (10)	0.0002 (9)
C6	0.0308 (11)	0.0234 (11)	0.0311 (12)	−0.0045 (9)	0.0016 (9)	−0.0091 (9)
C7	0.0209 (10)	0.0218 (10)	0.0221 (10)	−0.0044 (8)	−0.0010 (8)	−0.0068 (8)
C8	0.0267 (10)	0.0217 (10)	0.0207 (10)	−0.0047 (8)	0.0021 (8)	−0.0087 (8)
C9	0.0321 (11)	0.0234 (10)	0.0238 (11)	−0.0020 (9)	−0.0065 (8)	−0.0059 (8)
C10	0.0378 (12)	0.0290 (11)	0.0186 (10)	−0.0061 (9)	−0.0038 (8)	−0.0097 (8)
C11	0.0241 (11)	0.0248 (11)	0.0333 (12)	−0.0057 (9)	−0.0026 (9)	−0.0075 (9)

### Geometric parameters (Å, °)

**Table:**

Ni1—O1^i^	2.0695 (14)	C2—C3	1.385 (3)
Ni1—O1	2.0695 (14)	C2—C7	1.399 (3)
Ni1—N1^i^	2.0908 (16)	C3—C4	1.371 (3)
Ni1—N1	2.0908 (16)	C3—H3	0.9300
Ni1—O1W	2.1036 (16)	C4—C5	1.395 (3)
Ni1—O1W^i^	2.1036 (16)	C4—H4	0.9300
N1—C1	1.307 (2)	C5—C6	1.373 (3)
N1—C7	1.395 (2)	C5—H5	0.9300
N2—C1	1.354 (2)	C6—C7	1.389 (3)
N2—C2	1.391 (2)	C6—H6	0.9300
N2—C8	1.424 (2)	C8—C9	1.377 (3)
O1—C11	1.245 (2)	C8—C10^ii^	1.385 (3)
O2—C11	1.236 (2)	C9—C10	1.380 (3)
O2W—H2A	0.8522	C9—H9	0.9300
O2W—H2B	0.8516	C10—C8^ii^	1.385 (3)
O1W—H1A	0.8504	C10—H10	0.9300
O1W—H1B	0.8516	C11—H11	0.9300
C1—H1	0.9300		
			
O1^i^—Ni1—O1	180.00 (5)	C3—C2—C7	122.24 (18)
O1^i^—Ni1—N1^i^	87.66 (6)	N2—C2—C7	105.25 (16)
O1—Ni1—N1^i^	92.34 (6)	C4—C3—C2	116.98 (19)
O1^i^—Ni1—N1	92.34 (6)	C4—C3—H3	121.5
O1—Ni1—N1	87.66 (6)	C2—C3—H3	121.5
N1^i^—Ni1—N1	180.00 (10)	C3—C4—C5	121.4 (2)
O1^i^—Ni1—O1W	94.56 (6)	C3—C4—H4	119.3
O1—Ni1—O1W	85.44 (6)	C5—C4—H4	119.3
N1^i^—Ni1—O1W	89.72 (6)	C6—C5—C4	121.7 (2)
N1—Ni1—O1W	90.28 (6)	C6—C5—H5	119.2
O1^i^—Ni1—O1W^i^	85.44 (6)	C4—C5—H5	119.2
O1—Ni1—O1W^i^	94.56 (6)	C5—C6—C7	117.75 (19)
N1^i^—Ni1—O1W^i^	90.28 (6)	C5—C6—H6	121.1
N1—Ni1—O1W^i^	89.72 (6)	C7—C6—H6	121.1
O1W—Ni1—O1W^i^	180.00 (11)	C6—C7—N1	130.60 (17)
C1—N1—C7	105.03 (15)	C6—C7—C2	119.93 (18)
C1—N1—Ni1	120.95 (13)	N1—C7—C2	109.45 (16)
C7—N1—Ni1	133.87 (12)	C9—C8—C10^ii^	120.26 (18)
C1—N2—C2	106.47 (16)	C9—C8—N2	120.29 (18)
C1—N2—C8	124.79 (17)	C10^ii^—C8—N2	119.45 (17)
C2—N2—C8	128.57 (16)	C8—C9—C10	119.76 (19)
C11—O1—Ni1	123.29 (13)	C8—C9—H9	120.1
H2A—O2W—H2B	109.0	C10—C9—H9	120.1
Ni1—O1W—H1A	124.7	C9—C10—C8^ii^	119.98 (18)
Ni1—O1W—H1B	114.7	C9—C10—H10	120.0
H1A—O1W—H1B	105.8	C8^ii^—C10—H10	120.0
N1—C1—N2	113.79 (18)	O2—C11—O1	126.04 (19)
N1—C1—H1	123.1	O2—C11—H11	117.0
N2—C1—H1	123.1	O1—C11—H11	117.0
C3—C2—N2	132.49 (18)		
			
O1^i^—Ni1—N1—C1	−139.94 (16)	N2—C2—C3—C4	178.9 (2)
O1—Ni1—N1—C1	40.06 (16)	C7—C2—C3—C4	0.7 (3)
N1^i^—Ni1—N1—C1	152 (100)	C2—C3—C4—C5	−1.2 (4)
O1W—Ni1—N1—C1	−45.36 (16)	C3—C4—C5—C6	0.4 (4)
O1W^i^—Ni1—N1—C1	134.64 (16)	C4—C5—C6—C7	0.8 (3)
O1^i^—Ni1—N1—C7	45.25 (18)	C5—C6—C7—N1	−179.6 (2)
O1—Ni1—N1—C7	−134.75 (18)	C5—C6—C7—C2	−1.2 (3)
N1^i^—Ni1—N1—C7	−23 (100)	C1—N1—C7—C6	178.2 (2)
O1W—Ni1—N1—C7	139.83 (18)	Ni1—N1—C7—C6	−6.4 (3)
O1W^i^—Ni1—N1—C7	−40.17 (18)	C1—N1—C7—C2	−0.3 (2)
O1^i^—Ni1—O1—C11	90 (100)	Ni1—N1—C7—C2	175.08 (14)
N1^i^—Ni1—O1—C11	−48.98 (16)	C3—C2—C7—C6	0.5 (3)
N1—Ni1—O1—C11	131.02 (16)	N2—C2—C7—C6	−178.12 (18)
O1W—Ni1—O1—C11	−138.51 (16)	C3—C2—C7—N1	179.16 (19)
O1W^i^—Ni1—O1—C11	41.49 (16)	N2—C2—C7—N1	0.6 (2)
C7—N1—C1—N2	−0.1 (2)	C1—N2—C8—C9	−145.1 (2)
Ni1—N1—C1—N2	−176.23 (13)	C2—N2—C8—C9	40.3 (3)
C2—N2—C1—N1	0.5 (2)	C1—N2—C8—C10^ii^	35.2 (3)
C8—N2—C1—N1	−175.17 (18)	C2—N2—C8—C10^ii^	−139.5 (2)
C1—N2—C2—C3	−179.0 (2)	C10^ii^—C8—C9—C10	−0.3 (3)
C8—N2—C2—C3	−3.6 (4)	N2—C8—C9—C10	179.95 (18)
C1—N2—C2—C7	−0.6 (2)	C8—C9—C10—C8^ii^	0.3 (3)
C8—N2—C2—C7	174.80 (18)	Ni1—O1—C11—O2	−169.69 (16)

Symmetry codes: (i) −*x*+2, −*y*, −*z*; (ii) −*x*+1, −*y*+1, −*z*+1.

### Hydrogen-bond geometry (Å, °)

**Table:**

*D*—H···*A*	*D*—H	H···*A*	*D*···*A*	*D*—H···*A*
O1W—H1A···O2^iii^	0.85	1.85	2.694 (2)	169
O1W—H1B···O2W^iv^	0.85	1.92	2.762 (2)	169
O2W—H2A···O2^v^	0.85	1.91	2.760 (2)	173
O2W—H2B···O1^vi^	0.85	2.16	2.846 (2)	137

Symmetry codes: (iii) *x*−1, *y*, *z*; (iv) *x*, *y*, *z*−1; (v) −*x*+2, −*y*+1, −*z*+1; (vi) *x*, *y*, *z*+1.
